# Geographic Tongue and Fissured Tongue in 348 Patients with Psoriasis: Correlation with Disease Severity

**DOI:** 10.1155/2015/564326

**Published:** 2015-01-19

**Authors:** Bruna L. S. Picciani, Thays T. Souza, Vanessa de Carla B. Santos, Tábata A. Domingos, Sueli Carneiro, João Carlos Avelleira, David R. Azulay, Jane M. N. Pinto, Eliane P. Dias

**Affiliations:** ^1^Hospital Universitário Antonio Pedro, Faculdade de Medicina, Departamento de Patologia, Universidade Federal Fluminense, Rua Marques de Parana 303, 4° Andar, 24033-900 Niteroi, Rio de Janeiro, RJ, Brazil; ^2^Sector of Dermatology, Rio de Janeiro Federal University, Rio de Janeiro, Brazil; ^3^Department of Dermatology Rubem David Azulay, Santa Casa da Misericórdia, Rio de Janeiro, Brazil; ^4^Department of Dermatology, Universidade Federal Fluminense, Rio de Janeiro, Brazil

## Abstract

Geographic tongue (GT) and fissured tongue (FT) are the more frequent oral lesions in patients with psoriasis. The aims of this study were to compare the prevalence of GT/FT between psoriasis group (PG) and healthy controls (HC) and investigate the correlation between GT/FT and psoriasis severity using the PASI and age of psoriasis onset. Three hundred and forty-eight PG and 348 HC were selected. According to the age of psoriasis onset, the individuals were classified as having early psoriasis and late psoriasis. The severity of vulgaris psoriasis was determined according to PASI. A follow-up was conducted in patients with psoriasis vulgaris (PV) with GT to evaluate the progression of oral and cutaneous lesions. The FT and GT were more frequent in PG than in HC. The incidence of GT was higher in patients with early psoriasis and that of FT in late-psoriasis. There is association between psoriasis intensity and GT; and a higher monthly decrease of PASI score in patients without GT. The presence of GT and FT is higher in PG than in the HC. GT is associated with disease severity and may be a marker of the psoriasis severity.

## 1. Introduction

Psoriasis is a common inflammatory cutaneous disease affecting 1%–3% of the world population [1]. The presence of oral lesions in psoriasis is uncommon and controversial [2–5]. Some reports have stated that oral lesions do not occur in psoriasis [6], and some have reported that they rarely occur [7, 8]. The oral manifestations of psoriasis mostly include nonspecific lesions such as geographic tongue (GT) and fissured tongue (FT). In addition, it has been also considered that these conditions are associated with psoriasis [2–4]. GT presents with clinical, histological, and genetic patterns similar to those of psoriasis, suggesting that this lesion may represent an oral manifestation of psoriasis [3, 4, 9, 10]. However, some authors have questioned this because GT usually appears without cutaneous lesions [8, 11, 12]. Gonzaga and Consolaro showed that GT may be a failed form of psoriasis that proceeds with or without subsequent simultaneity [13]. A positive family history, an association between psoriasis and GT, and the presence of the histocompatibility antigen HLA-Cw6 suggest a genetic basis, providing further evidence that these disorders are related [10]. Moreover, increased GT occurrence in patients with severe psoriasis vulgaris suggests that this lesion may be a marker of psoriasis severity [14–16]. This study aimed to compare the prevalence of GT/FT between PG and healthy controls (HC) and investigate the correlation between GT/FT and psoriasis severity using the Psoriasis Area and Severity Index (PASI) and age of psoriasis onset.

## 2. Material and Methods

This study included 348 Brazilian PG treated at three dermatology services (Universidade Federal Fluminense, Universidade Federal do Rio de Janeiro, and Santa Casa de Misericórdia) and 348 HC recruited from among potential bone marrow donor volunteers and from the Oral Medicine Service of Universidade Federal Fluminense. The protocol was approved by the Ethics Committee, and an informed consent form was signed by each participant. The exclusion criteria included an age of <18 years, hospitalization, and the absence of clinical data in the institution's records. HC with a current or past history of any dermatological disease were also excluded. Data on demographic characteristics, habits, medical history, and relevant information on the disease were collected and, according to the age of psoriasis onset, PG were classified as having early (starting before or at the age of 30) or late psoriasis (after the age of 30). The severity of psoriasis vulgaris (PV) was determined according to PASI score; a PASI score of >12 was defined as being severe, that of 7–12 was defined as being moderate, and that of <7 was defined as being mild chronic plaque-type psoriasis. The PASI score was used only for classifying psoriasis vulgaris because other clinical forms are generally considered to be severe and do not fit the criteria used by the PASI [17]. All participants underwent an oral mucosal examination performed by an examiner (B. L. S. Picciani) who used artificial light, gloves, and a wooden spatula for the evaluation of oral soft tissues. The diagnosis criterion of GT was based on the presence of red areas surrounded by a yellow-white border and FT was based on the presence of fissures. Scrapings from both lateral borders and the dorsal surface of the tongue were obtained using a sterile cytobrush (Kolplast, Brazil) to rule out candidiasis. PV and GT were followed up with PASI evaluations and oral examinations at two visits quarterly to evaluate the progression of simultaneous oral and cutaneous lesions. SPSS version 17.0 for Windows (SPSS Inc., Chicago, IL, USA) was used for all statistical analyses. Numerical variables are expressed as means ± standard deviations. Categorical variables are expressed as absolute (*N*) and relative (%) frequencies. In addition, the odds ratio (OR) was calculated with a 95% confidence interval (CI). Statistical analysis included Fisher's exact test for categorical variables and the Mann-Whitney or Kruskal-Wallis test for numerical variables. A *P*  value of <0.05 was considered statistically significant.

## 3. Results

**Figure 1:**
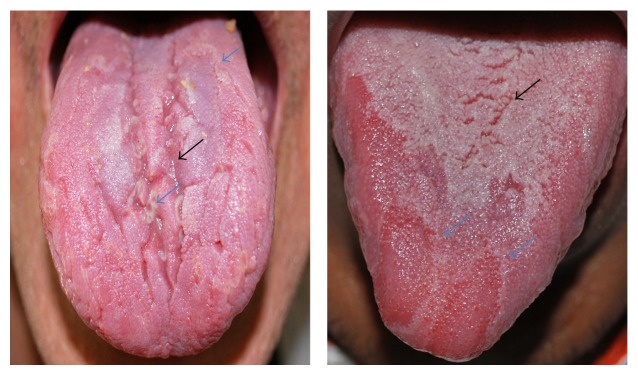
Fissured tongue and geographic tongue. Clinical aspects of fissured tongue (black arrow) and geographic tongue (blue arrow).

**Figure 2:**
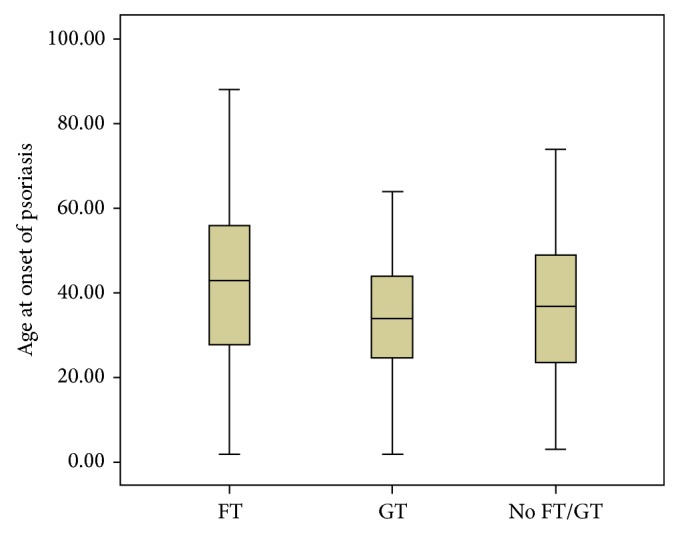
Box plot showing the distribution of psoriatic patients with fissured tongue, geographic tongue, and without tongue lesions, according to age at onset of psoriasis. Kruskal-Wallis test, *P* = 0.004; Mann-Whitney test, FT × GT, *P* = 0.008.

**Figure 3:**
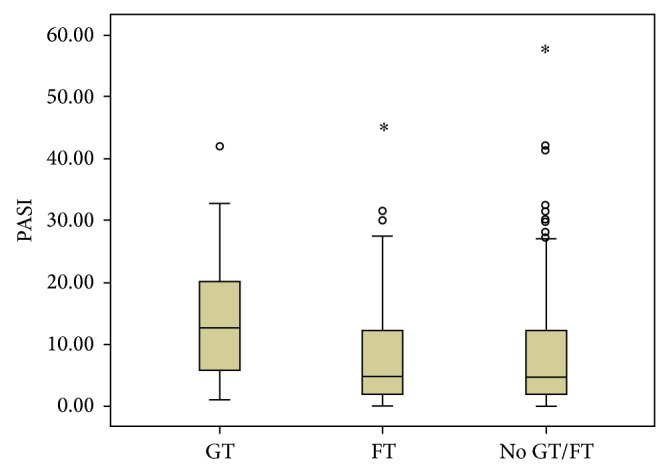
Box plot showing the distribution of PASI scores in the groups studied. Kruskal-Wallis test, *P* ≤ 0.001; Mann-Whitney test, GT × FT and GT × No GT/FT, *P* ≤ 0.001.

The PG group included 348 patients (177 (51%) women; 181 (52%) Caucasians; average age, 51 ± 15 years; range, 18–90 years). The HC group included 348 individuals (195 (55%) women; 232 (66%) Caucasians; average age, 46 ± 19 years; range, 18–90 years). Psoriasis vulgaris (81%) was the most common clinical type observed in this cohort. The mean age at diagnosis was 38 ± 16.3 years (range, 6–78 years). Early-onset psoriasis was observed in 131 (38%) patients. Totally, 184 (53%) patients were receiving systemic therapy, 125 (36%) were receiving topical treatment, 25 (7%) were receiving phototherapy (narrow-band ultraviolet B), and 14 (4%) were not receiving any therapy. The severity of chronic plaque-type psoriasis as assessed by PASI scores was mild in 157 (55%) patients, moderate in 43 (15%), and severe in 84 (30%). The GT (Figure 1) was more frequent in PG than in HC, with 43 (12%) and 10 (3%) patients, respectively (*P* = 0.002; OR = 4.76; 95% CI: 2.30–10.8; Table 1). The FT (Figure 1) was detected more frequently in PG than HC, with 125 (36%) and 70 (20%) patients (*P* ≤ 0.001; OR = 3.45; 95% CI: 2.44–4.91; Table 1). The incidence of GT (65%) was higher in patients with early-onset psoriasis and FT (58%) had higher frequency in patients with late psoriasis (Table 2). The Kruskal-Wallis test indicated a statistically significant difference between the groups with regard to the age of psoriasis onset (*P* = 0.004). The box plot shows the distribution of the age of psoriasis onset in the groups studied (Figure 2). With regard to the severity of psoriasis vulgaris, the PASI scores were mild in 88 (59%) PV, 61 (61%) FT, and 8 (22%) GT; moderate in 23 (16%) PV, 13 (13%) FT, and 7 (20%) GT; and severe in 37 (25%) PV, 26 (26%) FT, and 21 (58%) GT. A Kruskal-Wallis test indicated a statistically significant difference among the groups in relation to the PASI score (*P* ≤ 0.001). The Mann-Whitney test showed a statistically significant association (*P* < 0.001) between psoriasis severity and GT occurrence (Figure 3). The considered patients with severe psoriasis vulgaris, as assessed by PASI, with GT showed proportional rates that were twofold higher than those shown by PV without GT (25% versus 58%, resp.). The box plot shows the distribution of PASI scores in the studied groups (Figure 3). Considering the 36 patients with GT and psoriasis vulgaris, 22 (61%) returned for further appointments, while 11 (50%) had no oral lesions. The average monthly PASI score in three months was −8.7 points (standard derivation 9.8 points) in patients without GT and 0.7 points (standard derivation 4.1 points) in patients with GT (*t* test, *P* = 0.014; Table 3). The average monthly PASI score in six months was −11.6 points (standard derivation 9.1 points) in patients without GT and 1.1 points (standard derivation 8.7 points) in patients with GT (*t* test, *P* = 0.0003; Table 3).

## 4. Discussion

The oral manifestations of psoriasis were first described by Oppenheim in 1903, but nowadays the occurrence of specific oral lesion has been questioned [17, 22]. The clinical features of oral psoriasis are great variable and may affect any site of the oral mucosa, making the diagnosis a difficulty [22]. Several oral lesions have been described, as white or grey plaques to annular lesions, diffuse areas of erythema, and nonspecific lesions as geographic tongue [7, 14]. In this study, with a large cohort of psoriatic patients, any specific oral psoriasis lesion was not found. Fissured tongue (FT) and geographic tongue (GT) are considered the most common oral lesions in patients with psoriasis, comprising a range of nonspecific lesions [16, 19]. Different studies have found the prevalence of FT and GT to be 6–33% and 1–18% (Table 4), respectively [2, 4, 14, 18, 20, 21, 23–25]. These findings are similar to our results, in which a higher frequency of GT and FT in psoriatic patients (12% and 36%) than in healthy controls (3% and 20%) was detected. According to Daneshpazhooh et al. [14], FT occurred in 33% of the patients with psoriasis and GT in 14% of them, compared to 9.5% and 6% in the control group. In another controlled study, FT and GT were present in 47.5% and 12.5% of psoriatic patients and 20.4% and 4.7% of control individuals, respectively [18]. Some studies have suggested that GT and FT appeared at higher frequency in generalized pustular psoriasis [14, 26]. However, the analysis of this association was limited in the present and other studies, due to the small number of pustular psoriasis cases. Dawson [27] suggests the possibility of a genetic link between psoriasis and GT or/and FT. This association is reinforced by the fact that 35% of patients with psoriasis have a positive family history [8], a feature highlighted by our results that showed 42.4% of our patients with a familial history of psoriasis. The positive family history and the association of both psoriasis and GT with HLA-Cw6 suggest a genetic basis for this association and provide further evidence that the two disorders are related [10]. The significance of the higher frequency of FT in psoriatic patients was not established and FT is considered to be a genetically inherited trait [2]. Gonzaga et al. [28] showed that HLA-C^*^06 was not associated with FT, reinforcing the hypothesis that there is not any common genetic factor among GT, FT, and psoriasis. When associated with GT, the FT may be a sequel. The frequency of GT is increased in the severity of psoriasis, and it may be considered a marker of disease severity (Table 4). However, within our study, the frequency of FT did not increase by increasing severity of psoriasis [14]. Pogrel and Cram [29] suggest that patients with acute exacerbation of psoriasis develop more oral lesions than the patients with chronic stable disease. Singh et al. [16] showed that patients with GT had more severe psoriasis compared to those without tongue lesions. Psoriasis severity was assessed by the age of psoriasis onset and the PASI score. PASI is the most widely used score system in this disease. In relation to the age of psoriasis onset, our data showed a statistically significant occurrence of GT in early-onset psoriasis, whereas FT was associated with late-onset psoriasis. Ulmansky et al. [11] suggested that GT is a psoriatic expression of a transitory character, whereas FT may be a later, more permanent expression of psoriasis. The early-onset psoriasis is considered more severe and is associated with HLA-C^*^06 when compared with late-onset psoriasis [30, 31]. There are different definitions for the age of psoriasis onset, making the comparisons a difficulty. In this study, it was considered 30 years old to classify the two types of psoriasis, based on the clinical dermatologic experience of the authors. The authors believe that patients at the age of onset less than or equal to 30 years definitely have early-onset psoriasis [31]. Picciani et al. [4] found all cases of GT in early psoriasis and Zargari [15] showed that GT was higher in early-onset than in late-onset psoriasis (7.2% versus 1.3%), although both studies were not statistically significant (*P* = 0.008 and *P* = 0.056, resp.). There are few reports showing the relationship between the presence of GT and PASI score. Daneshpazhooh et al. [14] reported that 19.3% of the psoriatic patients without GT and 32.1% of the psoriatic patients with GT were included in the severe category, suggesting that the frequency of GT increased with the severity of psoriasis. Singh et al. [16] analyzed PASI as a continuous variable and it was demonstrated that patients with GT had more severe psoriasis compared to those patients who did not have GT (*P* ≤ 0.001). Similar to these studies, our results showed a statistically significant association between psoriasis severity and the presence of GT (*P* ≤ 0.001). This fact (the association between psoriasis severity and the presence of GT) suggests that GT may be a marker of the psoriasis severity. The present study was the first to perform patients' follow-up and the monitoring of oral and cutaneous lesions through oral exam and PASI. Our findings showed a higher decrease of PASI score in patients without GT compared to patients with GT. A limitation to this analysis was that few patients returned for follow-up, and it was difficult to investigate the development of oral and cutaneous lesions. Considering only one oral exam it may decrease the prevalence of oral lesions, because patients present remission and exacerbation periods, especially in mild psoriasis.

## 5. Conclusion

In conclusion, the present study showed that GT and FT are higher in psoriatic patients than in the general population. GT was more common in early-onset psoriasis and it is associated with disease severity. FT occurred with more frequency in late psoriasis, supporting that it can be a permanent consequence of GT. Geographic tongue is a marker of the psoriasis severity and its presence may be used as an additional criterion for disease severity. Moreover, to improve our understanding, oral exam should be performed in the routine medical evaluation of psoriatic patients.

## Figures and Tables

**Table 1:** Prevalence of fissured tongue and geographic tongue in patients with psoriasis (PG) and healthy controls (HC).

Oral lesion	PG *n* = 348 (%)	HC *n* = 348 (%)	Odds ratio (95% CI)	*P* ^*^
Fissured tongue	125 (36%)	70 (20%)	3.45 (2.44–4.91)	<0.001
Geographic tongue	43 (12%)^**^	10 (3%)^**^	4.76 (2.30–10.8)	0.002

^*^Fisher's exact test, 2-tailed; CI: confidence interval.

^**^80% of patients had both geographic tongue and fissured tongue.

**Table 2:** Distribution of 348 psoriatic patients with and without tongue lesions according to age at onset of psoriasis.

Age at onset of psoriasis (years)	FT	GT	Total patients without tongue lesions	Total
Early-onset	52 (42%)	28 (65%)	98 (54%)	178
Late-onset	73 (58%)	15 (35%)	82 (46%)	170
Total	**125**	**43**	**180**	**348**

FT—fissured tongue; GT—geographic tongue.

**Table 3:** Distribution of 22 psoriatic patients with geographic tongue according to distribution of PASI variation rate and evaluation of oral lesion.

Cases	Psoriasis treatment	1° PASI	2° PASI 3 months	PASI variation rate 1	GT	3° PASI 6 months	GT	PASI variation rate 2
01	Systemic	32	32.3	0.3	Yes	8.2	No	−23.8
02	Topical	32.6	29.1	−3.5	Yes	30.1	Yes	−2.5
03	Systemic	42	41.5	−0.5	Yes	27.3	Yes	−14.7
04	Systemic	1.3	0.6	−0.7	No	0	No	−1.3
05	Topical	1.5	1.5	0	No	0.5	No	−1
06	Systemic	3.6	12.9	9.3	Yes	19.2	Yes	15.6
07	Systemic	7	3.6	−3.4	No	0	No	−7
08	Topical	21.9	19.5	−2.4	Yes	14.4	Yes	−7.5
09	Topical	27	7	−20	No	9.6	No	−17.4
10	Systemic	3.6	4.6	1	Yes	17.4	Yes	13.8
11	Topical	22	16.5	−5.5	Yes	7.4	No	−14.6
12	Topical	17.6	14.7	−2.9	Yes	19.2	Yes	1.6
13	Topical	18.2	20.5	2.3	Yes	23.3	Yes	5.1
14	Systemic	31	3.6	−27.4	No	1.5	No	−29.5
15	Topical	2.5	2	−0.5	Yes	6	Yes	3.5
16	Topical	12	8	−4	No	5.4	No	−6.6
17	Topical	13.6	18.2	4.6	Yes	13.3	Yes	−0.3
18	Systemic	16.5	7	−9.5	No	4	No	−12.5
19	Systemic	13.3	11.8	−1.5	Yes	15.1	Yes	1.8
20	Systemic	8	3.2	−4.8	No	2.5	No	−5.5
21	Systemic	13.6	9.5	−4.1	Yes	5.4	No	−8.2
22	Topical	31	24.7	−6.3	Yes	27	Yes	−4

GT—geographic tongue.

**Table 4:** Prevalence of geographic tongue (GT) and fissured tongue (FT) in patients with psoriasis (P) and healthy controls (C) and association with age of psoriasis onset and psoriasis severity (PASI).

References	Total (*N*)		GT (%)		FT (%)		GT X psoriasis onset (%)		FT X psoriasis onset (%)		GT X PASI (%)			FT X PASI (%)		
	P	C	P	C	P	C	Early-onset	Late-onset	Early-onset	Late-onset	Mild	Moderate	Severe	Mild	Moderate	Severe
Daneshpazhooh et al. (2004) [14]	200	200	14	6	33	9.5	No^*^	No^*^	No^*^	No^*^	5.7	16.7	32.1	26.4	38.3	25
Zargari (2006) [15]	306	No^*^	7.2	No^*^	9.8	No^*^	7.2^**^	1.3	8.2	11.8	No^*^	No^*^	No^*^	No^*^	No^*^	No^*^
Hernandez-Perez et al. (2008) [18]	80	127	12.5	4.7	47.5	20.4	No^*^	No^*^	30.4^***^	54.4	No^*^	No^*^	No^*^	No^*^	No^*^	No^*^
Costa et al. (2009) [2]	166	166	18.1	4.2	34.3	16.2	No^*^	No^*^	No^*^	No^*^	No^*^	No^*^	No^*^	No^*^	No^*^	No^*^
Tomb et al. (2010) [19]	400	1000	7.7	1	33.2	9.9	No^*^	No^*^	No^*^	No^*^	No^*^	No^*^	No^*^	No^*^	No^*^	No^*^
Picciani et al. (2011) [4]	203	No^*^	12.1	No^*^	34.4	No^*^	100^***^	0	38	62	No^*^	No^*^	No^*^	No^*^	No^*^	No^*^
Germi et al. (2012) [20]	535	436	9.1	5.2	22.6	10.3	No^*^	No^*^	No^*^	No^*^	No^*^	No^*^	No^*^	No^*^	No^*^	No^*^
Darwazeh et al. (2012) [21]	100	100	17	9	35	13	20.8^**^	7.1	29	50	19.7	5.6	40	36.1	22.2	50
Singh et al. (2013) [16]	600	800	5.6	0.8	45.3	40	5.4^***^	6.2	No^*^	No^*^	No^*^	No^*^	12.9^****^	No^*^	No^*^	No^*^

No^*^= there is no information; P = psoriasis; C = control; GT = geographic tongue; FT = fissured tongue; ^**^early-onset = ≤30 years; ^***^early-onset = ≤40 years.

^****^This value represents the average of PASI found in psoriasis patients with geographic tongue.

## Abbreviations

PG: Psoriasis group
PV: Psoriasis vulgaris
GT: Geographic tongue
FT: Fissured tongue
HC: Healthy controls
PASI: Psoriasis Area and Severity Index.
